# Expression of ubiquitin carboxyl-terminal hydrolase isozyme L1 and chromogranin A regulated via histone-modification through Rho/ERK/NFκB signaling in oxaliplatin-resistant colorectal cancer cells

**DOI:** 10.7150/ijms.126460

**Published:** 2026-02-26

**Authors:** Ko-Chao Lee, Kung-Chuan Cheng, Cheng-Yi Huang, Meng-Chiao Hsieh, Shui-Yi Tung, Yung-Yu Hsieh, Kam-Fai Lee, Chih-Chuan Teng, Hsing-Chun Kuo

**Affiliations:** 1 Division of Colorectal Surgery, Department of Surgery, Kaohsiung Chang Gung Memorial Hospital, Chang Gung University College of Medicine, Kaohsiung, Taiwan.; 2 Division of Colorectal Surgery, Department of Surgery, Kaohsiung Municipal Fong Shan Hospital (Under the management of Chang Gung Medical Foundation), Kaohsiung, Taiwan.; 3 Division of Colorectal Surgery, Department of Surgery, Kaohsiung Municipal Ta-Tung Hospital, Kaohsiung, Taiwan.; 4 Division of Colorectal Surgery, Department of Surgery, Kaohsiung Chang Gung Memorial Hospital, Kaohsiung, Taiwan.; 5 Division of Colon and Rectal Surgery, Department of Surgery, Chang Gung Memorial Hospital, Chiayi, Taiwan.; 6 Division of Gastroenterology and Hepatology, Department of Internal Medicine, Chang Gung Memorial Hospital, Chiayi, Taiwan.; 7 College of Medicine, Chang Gung University, Taoyuan, Taiwan.; 8 Department of Pathology, Chang Gung Memorial Hospital, Chiayi, Taiwan.; 9 Department of Nursing, Division of Basic Medical Sciences, Chang Gung University of Science and Technology, Chiayi, Taiwan.; 10 Research Fellow, Chang Gung Memorial Hospital, Chiayi, Taiwan.; 11 Center for Drug Research and Development, Chang Gung University of Science and Technology, Taoyuan, Taiwan.; 12 Chronic Diseases and Health Promotion Research Center, Chang Gung University of Science and Technology, Chiayi, Taiwan.

## Abstract

**Background/Aims**: Globally, colorectal cancer ranks third in causing 900,000 deaths annually. Some patients undergoing chemotherapy develop resistance, leading to metastasis. We investigated CHGA and UCHL1 proteins correlate with lymph node metastasis (J Cell Mol Med. 2023;27:2004-2020). This study aimed to analyze the relationship of CHGA and UCHL1 with the epithelial-mesenchymal transition (EMT) and the Rho/ERK/NFκB signaling pathway in OXA-resistant CRC cells.

**Methods:**

Resistant colorectal cancer cells (HCT-116/OxR) were established using progressive exposure to oxaliplatin (OXA). We employed siRNA, western blot, ROS assessment, apoptosis, and cell cycle assays, and animal models, to examine histone modifications regulating CHGA and UCHL1, and their impact on chemoresistance.

**Results:**

HCT-116/OxR cells displayed significantly higher OXA tolerance (elevated IC_50_) and reduced apoptosis compared with parental HCT-116 cells, confirmed by MTT assays and DAPI staining. Silencing CHGA and UCHL1 genes effectively suppressed the mobility and invasiveness of OXA-resistant HCT-116/OxR cells while promoting G1 phase cell cycle arrest and reducing ROS production and intracellular calcium concentrations. Notably, targeted knockdown of CHGA and UCHL1 in HCT-116/OxR cells successfully restored OXA sensitivity and EMT markers and inactivation of Rho/ERK/NFκB pathway. Further *in vivo* validation demonstrated that the downregulation of CHGA and UCHL1 expression markedly attenuated OXA resistance in CRC cells. Both CHGA and UCHL1-activated transcription were regulated through the Rho/ERK/NF-κB signaling pathways by histone modifications of H3K4 trimethylation.

**Conclusions:**

In this study, Rho/ERK/NFκB signaling-mediated CHGA and UCHL1 expression, which is regulated through histone modifications and affects OXA-resistant CRC EMT outcomes, was assessed, and its potential as an early detection biomarker and prognostic indicator was explored with clinical applications.

## Introduction

Colorectal cancer (CRC) is one of the leading causes of death due to cancer globally, and is projected to increase by 60% by 2030, with annual estimates exceeding 2.2 million new cases and 1.1 million deaths 1. Research shows that over 60% of new cases are already at an advanced stage, with 22% presenting with distant metastasis 2. Current standard treatments of CRC include surgical resection combined with chemotherapy 3. When oxaliplatin (OXA) is used in combination with 5-fluorouracil and leucovorin (FOLFOX), the response rate for metastatic CRC exceeds 50%, with a median survival approaching 2 years 4. However, some patients with metastatic CRC eventually develop resistance to OXA, with a median time to disease progression of approximately 8 months 5. OXA resistance in CRC is closely associated with epithelial-mesenchymal transition (EMT), a critical mechanism in cancer progression 6. The Rho/ extracellular signal-regulated kinase/ nuclear factor κB (Rho/ERK/NFκB) signaling pathway can reciprocally activate and stabilize the EMT phenotype, collectively promoting the resistance of cancer cells to OXA 7,8. However, the precise mechanisms by which the Rho/ERK/NFκB signaling pathway and the proteins chromogranin A (CHGA) and ubiquitin carboxy-terminal hydrolase L1 (UCHL1) function in EMT require further investigation.

CHGA, a 48 kDa acidic glycoprotein in neuroendocrine cells 9, has multiple physiological functions, including granule formation, hormone storage, calcium regulation, and anti-inflammation 10. CHGA serves as a crucial biomarker for neuroendocrine tumors 11, with elevated plasma levels strongly correlating with tumor burden and poor overall survival 12-14. CHGA levels increase proportionally with disease progression, particularly correlated with lymph node metastasis 15,16. UCHL1 is an important deubiquitinating enzyme that plays a crucial role in the ubiquitin-proteasome system 17. Although UCHL1 was initially found to be expressed primarily in central and peripheral neurons, studies have revealed its abnormally high expression in various non-neuronal cancers, including CRC, breast cancer, and pancreatic cancer 16,18. In CRC, high expression of UCHL1 is significantly associated with poor prognosis and increased rates of tumor recurrence 19. UCHL1 promotes tumor cell invasion and metastasis by regulating β-catenin/T-cell factor (TCF) signaling pathways 20, and modulating EMT-related transcription factors such as Twist, leading to decreased E-cadherin and increased vimentin expressions 21. Despite these findings, the mechanism connecting CHGA and UCHL1 to OXA-resistant CRC cells remains to be clarified.

Building on our previous identification of UCH-L1 and CHGA as oncogenic proteins in CRC metastasis and LNM 16, we investigated mechanisms of chemoresistance through the Rho/ERK/NFκB signaling using OXA-resistant HCT-116/OxR cells. These cells showed significant chemoresistance compared to parental HCT-116 cells. Silencing CHGA and UCHL1 reduced cell migration, invasion, ROS generation, and calcium signaling, and induced arrest at the G1 phase. This intervention reversed OXA resistance by regulating the Rho/ERK/NF-κB and EMT pathways. *In vivo* studies confirmed that downregulation of CHGA and UCHL1 significantly reduced drug resistance, supporting their potential as CRC biomarkers.

## Materials and Methods

### Materials

All culture materials were purchased from Gibco (Grand Island, NY, USA). 3-(4,5-dimethylthiazol-2-yl)-2,5-diphenyltetrazoliumbromide (MTT), Intracellular calcium (Fluo-3am), 2,7-dichlorodihydrofluorescein diacetate (H_2_DCFDA), and Annexin V-FITC/PI apoptosis detection kit were purchased from Sigma (St. Louis, MO, USA). Mouse monoclonal antibodies against Cyclin D and Cyclin E were obtained from Santa Cruz Biotechnology (Santa Cruz, CA, USA), and anti-β-actin from Sigma-Aldrich (St. Louis, MO, USA). Rabbit polyclonal antibodies against CHGA and UCHL1 were from Bioss (Boston, MA, USA), while anti-β-catenin, anti-Twist, and anti-p-ERK1/2 (Thr202/Tyr204) were from GeneTex (Irvine, CA, USA). Anti-Vimentin and anti-p-Rho (Ser188) rabbit polyclonal antibodies were from Santa Cruz Biotechnology (Santa Cruz, CA, USA), respectively. Rabbit monoclonal antibodies against NFκB p105/p50 were from Cell Signaling Technology (Beverly, MA, USA). Santa Cruz Biotechnology supplied all RNA interference reagents.

### Cell culture and chemoresistance cell lines

Human colorectal cancer HCT-116 cells (CCL-247) were obtained from the American Type Culture Collection (ATCC) and cultured in RPMI medium (Gibco, CA, USA). Following established protocols 22, oxaliplatin-resistant cell lines (HCT-116/OxR) were created through a systematic exposure to increasing concentrations of oxaliplatin over four phases: starting with 0.1 μg/ml for six weeks, increasing to 0.5 μg/ml for eight weeks, followed by 1.0 μg/ml for another eight weeks, and finally culminating with 2.0 μg/ml for approximately ten weeks. The surviving cells from this selection process were designated as HCT-116/OxR and maintained in culture with 2.0 μg/ml oxaliplatin as previously described 5,22-26.

### Cell viability test and DAPI staining

HCT-116 and HCT-116/OxR cells followed by OXA treatment (0-135 μg/ml) for 24 hours. 3-(4,5-dimethylthiazol-2-yl)-2,5-diphenyltetrazolium bromide (MTT) solution (0.5 mg/mL) was used for cell viability analysis, as previously described 24. For nuclear staining, HCT-116 and HCT-116/OxR cells were treated with OXA for 24h. DAPI-stained cells were examined by fluorescence microscopy, as previously described 25. Apoptosis index was defined as the percentage of DAPI-stained cells showing nuclear condensation and fragmentation characteristic of apoptotic cell death.

### CHGA and UCHL1 siRNA transfection and establishment of stable drug-resistant cell lines

CHGA and UCHL1 gene silencing was achieved through siRNA transfection (sc-37212 and sc-42304-V respectively; control siRNA: sc-37007) with a 6-hour transfection period. Subsequently, stable knockdown cell lines were established by lentiviral transduction (sc-37212-V and sc-42305-V) in the presence of polybrene, followed by puromycin selection 27. Santa Cruz Biotechnology supplied all RNA interference reagents (Santa Cruz, CA, USA).

### Protein immunoblot analysis

Total protein was extracted in lysis buffer with protease inhibitors and quantified by BCA assay (Thermo). Proteins separated by 6-12% SDS-PAGE were transferred to PVDF membranes (Millipore), as previously described 28 After blocking, membranes were probed with primary antibodies overnight at 4°C (CHGA, UCHL1, β-catenin, Twist, Cyclin D1, Cyclin E, Vimentin, followed by secondary antibodies for 1h. Protein bands were visualized by ECL on MultiGel-21 system and quantified using ImageJ 1.50d (NIH).

### Migration and invasion assays

Cell migration and invasion were assessed by wound healing and Transwell assays. For migration, cells were seeded in culture-inserts (Blossom Biotechnologies) until confluence, then inserts were removed, and wound closure was monitored using Q.Capture software. For invasion, cells in serum-free medium were seeded in Matrigel-coated Transwell^®^ inserts (6.5mm, Corning). After 24h, invaded cells were fixed, dehydrated, stained with DAPI, and quantified in five random fields per well in triplicate.

### Intracellular ROS-DCFDA measure and calcium levels (Fluo-3am)

Intracellular ROS and calcium concentration were measured using fluorescent probes. ROS generation was detected using DCFDA (2',7'-dichlorofluorescein diacetate) (20 μM, 37°C, 1h) 29. Intracellular calcium [Ca²⁺] was monitored using Fluo-3/AM (10 µM, 37°C, 1h), as previously described 30.

### Flow cytometry detected apoptosis and cell cycle

Apoptosis was detected using Annexin V-FITC/PI double staining kit (Sigma-Aldrich), as previously described 31. For cell cycle analysis, transfected cells were fixed overnight in cold ethanol, stained with PI/RNase A solution, and analyzed by flow cytometry to determine cell cycle distribution and DNA content.

### *In vivo* studies

The study protocols for animal experimentation and care were approved by the Institutional Animal Care and Use Committee of Kaohsiung Chang Gung Memorial Hospital (IACUC approval: 2021062103). Animal models were created by injecting colorectal cancer cells (1 × 10^6^ in Matrigel^®^) into BALB/c-nu mice (4-6 weeks old, BioLASCO, TW). For resistance studies, oxaliplatin (5 mg/kg/day, intraperitoneally, for 5 days) was administered as pretreatment. After monitoring tumor development and body weight for 21-28 days, tumors were harvested for protein analysis 16,23.

### Chromatin Immunoprecipitation (ChIP) assay

Fix cells with 1% paraformaldehyde (15 min), wash with PBS. Lyse in SDS buffer (10 min, on ice). Sonicate chromatin to 300-500 bp fragments. Mix with ChIP dilution buffer and Salmon sperm DNA/Protein A agarose. Pre-clear (2h, 4°C), add primary antibody (12h), then add agarose (2h). Wash, elute, and reverse cross-link with NaCl (4h, 65°C). Treat with EDTA, Tris, and proteinase K (1h, 45°C). Purify DNA using ChIP DNA Clean & Concentrator. Perform qPCR using SYBR Green with cycling conditions: 95°C denaturation, 55-65°C annealing, 72°C extension. Amplify CHGA and UCHL1 promoter regions using specific primers were shown below:

CHGA promoter of primers (-1185 to -940 bp), Forward: 5'-CAGGCGTGAGCACAGGTGTG-3'; Reverse: 5'-CAGTTTCCTGGTTGGCTTCC-3'; UCHL1 promoter of primers (-407 to -230 bp), Forward: 5'-GGGGGCACACATTTACATTC-3'; Reverse: 5'-GAACACCCACCAACAAATCC-3'; Analyze data using % input (2^-ΔCt) and fold enrichment (2^ΔΔCt). Statistical significance: paired t-test, p< 0.05 16,23.

### Statistical analysis

Results are expressed as mean ± SD. Statistical analyses were performed using one-way ANOVA with post-hoc tests (SPSS v10.0). Differences were considered significant at p < 0.05 24.

## Results

### Establishment of HCT-116/OxR cells to distinguish cancer types, showing oxaliplatin resistance

**Figure 1:**
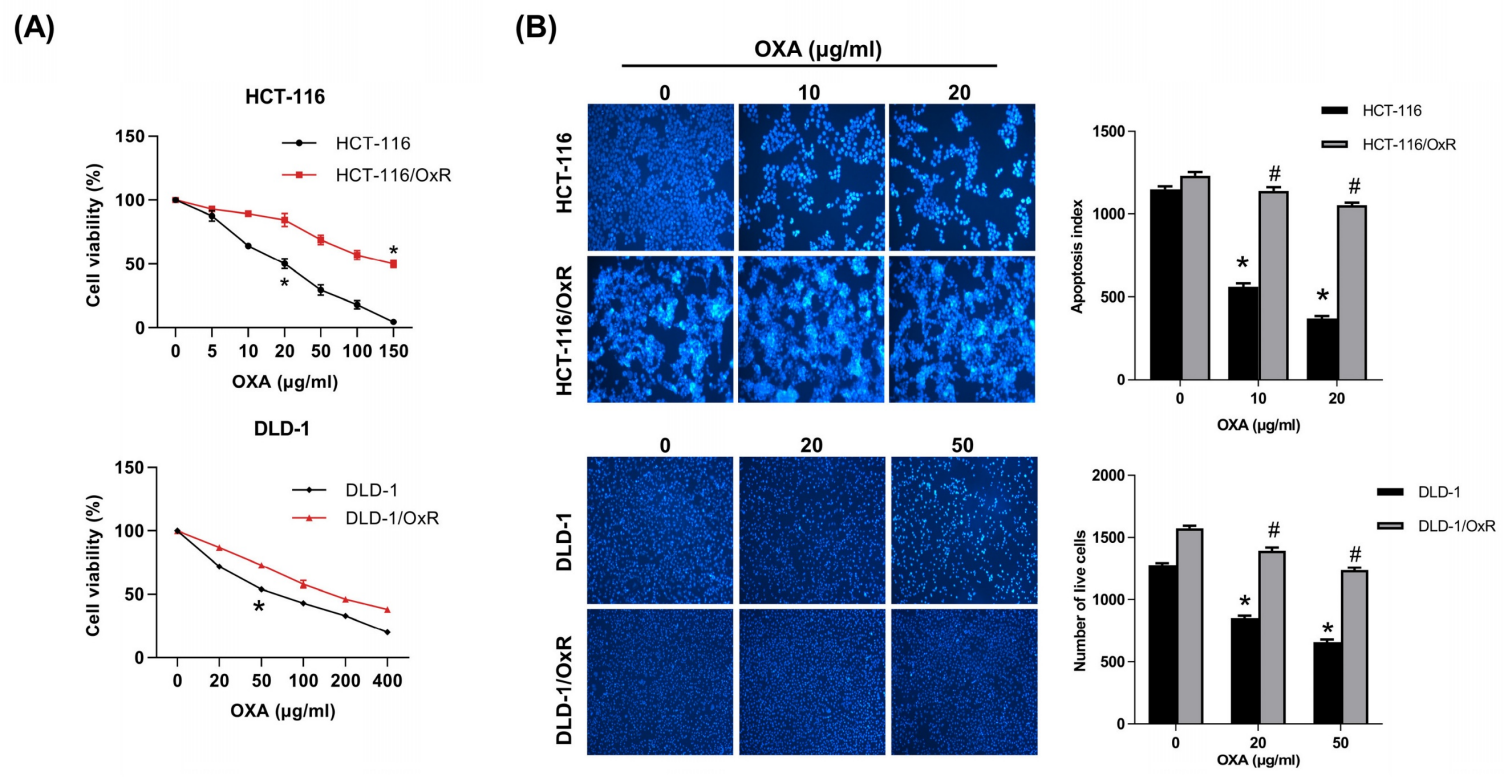
Establishment of chemoresistant HCT-116/OxR and DLD-1/OxR cell lines and exhibited decreased OXA-induced cell death. (A) The MTT assay was performed to evaluate the cytotoxicity of oxaliplatin (OXA) in two different cell lines: HCT-116 cells and their resistant variant, HCT-116/OxR cells as well as DLD-1 cells and their resistant variant, DLD-1/OxR cells. The IC_50_ (the concentration that inhibits 50% of cell growth) for OXA was determined to be 20 μg/ml in HCT-116 cells, while in HCT-116/OxR cells, it was found to be 150 μg/ml. (B) When administered at IC_50_ concentration, OXA reduced the number of live cells in both HCT-116 and HCT-116/OxR cell lines, as evidenced by DAPI staining. Results are presented as mean ± SD, with statistical significance set at *p < 0.05, versus the HCT-116 or DLD-1, untreated control group and ^#^p < 0.05, versus the HCT-116/OxR or DLD-1/OxR, untreated control group.

The CRC cell line (HCT-116 and DLD-1) and the drug-resistant CRC cell line (HCT-116/OxR and DLD-1/OxR) were employed to distinguish between CRC and drug-resistant CRC. Oxaliplatin (herein referred to as OXA) is a platinum-based anticancer medication used primarily for the treatment of CRC. Chemoresistant cells appeared elongated, exhibiting dyscohesive features typical of tumor-initiating cells, tumor-promoting cells, or cancer stem cells (CSC); western blotting and immunofluorescence analyses revealed that HCT-116/OxR cells expressed significantly higher levels of CSC markers (CD133 and CD44) and stemness factors (SOX2 and OCT4) than parental HCT-116 cells 25. In this study, OXA was selected as a probe drug to assess drug resistance characteristics. MTT analysis was performed to evaluate the differences between the HCT-116 cell line and its derived drug-resistant cell line, HCT-116/OxR and DLD-1/OxR. The half inhibitory concentration (IC_50_) measurement showed that the IC_50_ of HCT-116 was 20 and 50 μg/mL, while the IC_50_ of HCT-116/OxR and DLD/OxR were as high as 150 μg/mL (Figure 1A), indicating a significantly different response of HCT-116/OxR and DLD-1/OxR to the drug. Subsequently, the responses of the two cell lines to OXA were evaluated. The cells were treated with 10 μg/mL and 20 μg/mL as well as 20 μg/mL and 50 μg/mL OXA for 24 h, and changes in cell survival were observed using the DAPI staining method. Apoptosis index data showed that OXA treatment led to fewer viable HCT-116 cells compared with viable HCT-116/OxR or DLD-1/OxR cells (Figure 1B), confirming significant OXA resistance in HCT-116/OxR and DLD-1/OxR.

### Inhibition of chromogranin A and ubiquitin carboxyl-terminal hydrolase isozyme L1 expression impairs expansion and invasiveness in HCT-116/OxR cells

**Figure 2:**
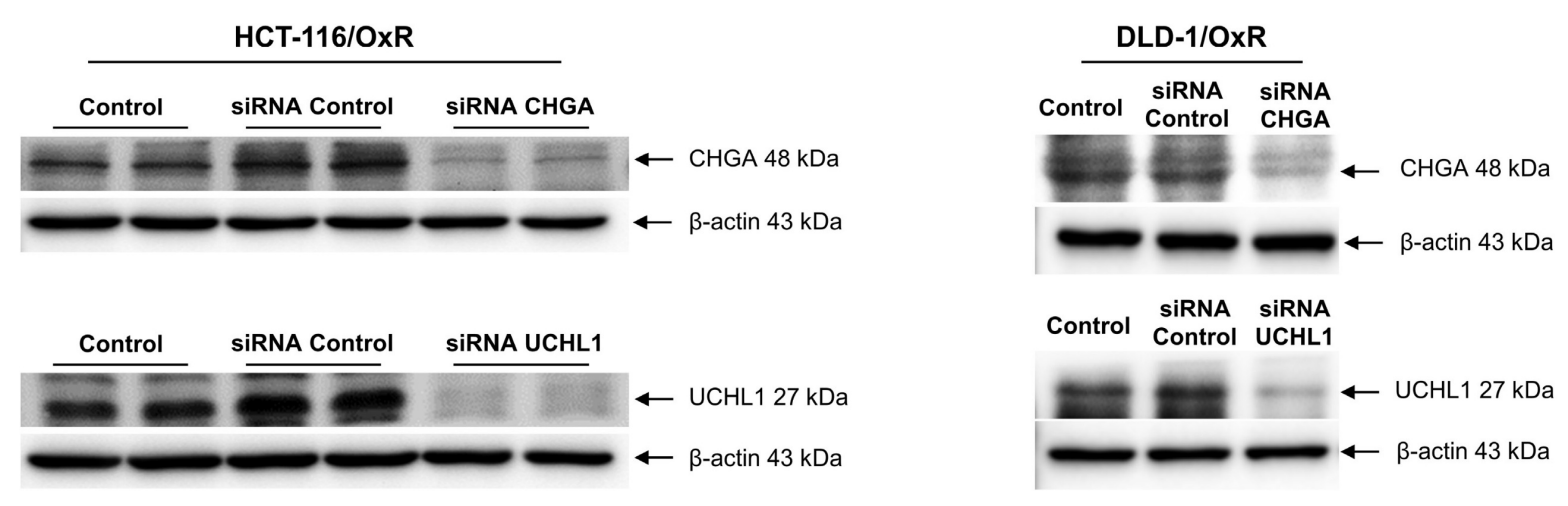
Expression of ubiquitin carboxyl-terminal hydrolase isozyme L1 and chromogranin A in chemoresistant HCT-116/OxR. Western blot analysis was performed using cell lysates from HCT-116/OxR cells, including untreated controls and cells transfected with control siRNA, CHGA siRNA, or UCHL1 siRNA.

**Figure 3:**
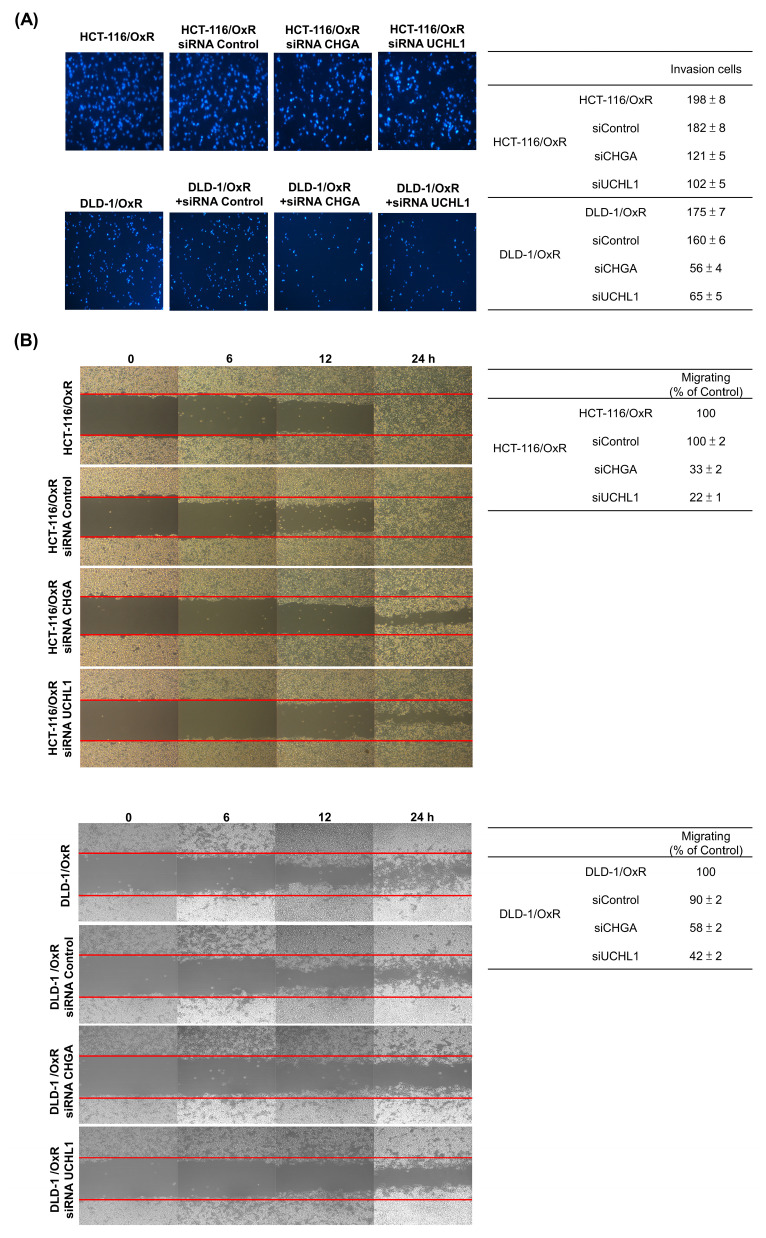
Loss functional analysis of ubiquitin carboxyl-terminal hydrolase isozyme L1 and chromogranin A expression on tumor growth, migration in HCT-116/OxR (A) HCT-116/OxR cells were transfected with three different siRNAs (CHGA, UCHL1, and control) for 6 hours, followed by culture medium replacement at 24 hours post-transfection. In a dual-chamber system, migrating cells through the inner membrane were observed and documented using microscopy at 200× magnification. Cell invasion was quantified by counting the number of cells that penetrated the inner membrane. Data are expressed as mean ± SD. (B) The drug-resistant cell line HCT-116/OxR received siRNA transfection treatment with siCHGA, siUCHL1, and control siRNA (siControl). Migration patterns were examined through wound healing analysis at 6, 12, and 24 hours post-transfection. Quantitative data are presented in tables, with the wound area of HCT-116/OxR cells normalized to 100% as the reference baseline.

CHGA and UCHL1 are important oncogenes. Their expression is closely associated with the occurrence of multiple cancers, including prostate cancer, esophageal cancer, and CRC. To investigate whether these two oncoproteins are involved in the drug resistance mechanism of the drug-resistant cell line HCT-116/OxR and DLD-1/OxR, siRNA technology was employed to knock down the CHGA and UCHL1 genes. The effects of reducing CHGA and UCHL1 expression on the CRC cells were observed, and their roles in the drug-resistant CRC cell line (HCT-116/OxR and DLD-1/OxR) were examined. The transfection of CHGA and UCHL1 siRNAs effectively reduces the protein expression of CHGA and UCHL1 in the HCT-116/OxR and DLD-1/OxR (Figure 2). To further understand the effect of CHGA and UCHL1 expression knockdown on the behavior of drug-resistant cancer cells, we used cell transwell and wound healing to evaluate the migration and invasion of HCT-116/OxR and DLD-1/OxR. The number of HCT-116/OxR and DLD-1/OxR cells that penetrated the membrane was 198 ± 8 and 175 ± 7, while that of the siRNA control group was 182 ± 8 and 160 ± 6. Accordingly, when the expression of CHGA and UCHL1 was inhibited, the number of HCT-116/OxR cells penetrating the membrane decreased to 121 ± 5 and 102 ± 5 (Figure 3A). The number of DLD-1/OxR cells penetrating the membrane decreased to 56 ± 4 and 65 ± 5. The results indicate that the inhibition of CHGA and UCHL1 expression effectively reduces the invasion capacity of cancer cells. Next, the migration ability of cancer cells was evaluated by observing cell healing at three time points: 6, 12, and 24 h. Considering the healing degree of untreated drug-resistant HCT-116/OxR and DLD-1/OxR cells as the standard (100%), the healing degree of CHGA and UCHL1 inhibition groups was reduced significantly to 33 ± 2% and 22 ± 1%, respectively. The healing degree of CHGA and UCHL1 inhibition groups was reduced significantly to 58 ± 2% and 42 ± 2%, respectively (Figure 3B). This result further confirmed that a decrease in the expression of CHGA and UCHL1 effectively inhibits the migration ability of cancer cells.

### Chromogranin A and ubiquitin carboxyl-terminal hydrolase isozyme L1 depletion modulated Ca²⁺ signaling, cell cycle, and oxidative balance, and restored sensitivity to OXA in HCT-116/OxR cells

**Figure 4:**
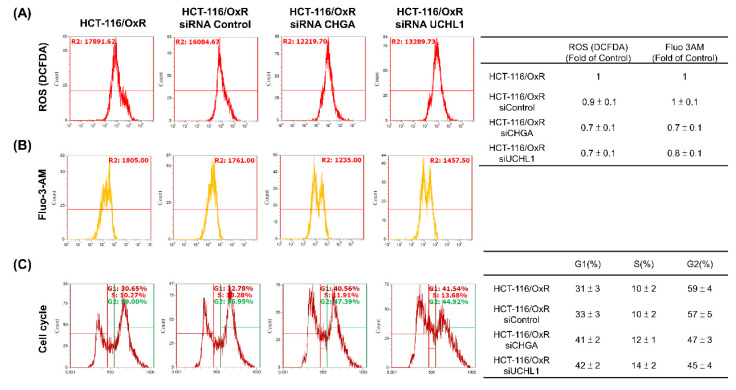
Loss functional analysis of ubiquitin carboxyl-terminal hydrolase isozyme L1 and chromogranin A expression on cell cycle checkpoint, intracellular calcium ions and release of reactive oxygen species in HCT-116/OxR (A and B) Flow cytometry analysis revealed alterations in reactive oxygen species, intracellular calcium ions, and cell cycle following CHGA and UCHL1 silencing in drug-resistant HCT-116/OxR cells. Post 24-hour gene knockdown, cellular samples underwent DCFDA and Fluo 3-AM staining. Measurements completed within 1 hour stain determined reactive oxygen species and intracellular calcium concentrations. Data presentations used the mean ± SD. Statistical comparisons of experimental samples referenced HCT-116/OxR baseline measurements. (C) Analysis of DNA content in HCT-116/OxR cells involved CHGA and UCHL1 suppression, followed by 24-hour alcohol fixation and propidium iodide staining. Cell cycle distribution percentages across G1, S, and G2/M phases underwent evaluation. Results expressed as mean ± SD.

The aforementioned experiments clearly indicate that the aggressiveness and metastatic potential of drug-resistant HCT-116/OxR cells can be reduced significantly by inhibiting the expression of CHGA and UCHL1. In CRC, elevated ROS levels and intracellular calcium signaling activate survival pathways that contribute to chemoresistance, concomitantly inhibiting apoptosis. Chemoresistant cells often bypass G1 phase arrest, maintaining proliferative capacity despite treatment. Based on this finding, the role of CHGA and UCHL1 in drug-resistant CRC cells was examined by using siRNA technology to inhibit the expression of these two genes and investigate their impact on cell cycle regulation. These results showed that in cells harboring siRNA CHGA and siRNA UCHL1, the content of intracellular ROS free radicals decreased significantly to 0.7 ± 0.1 and 0.7 ± 0.1, compared with the control HCT-116 /OxR cells as the baseline (set as 1) (Figure 4A).

**Figure 5:**
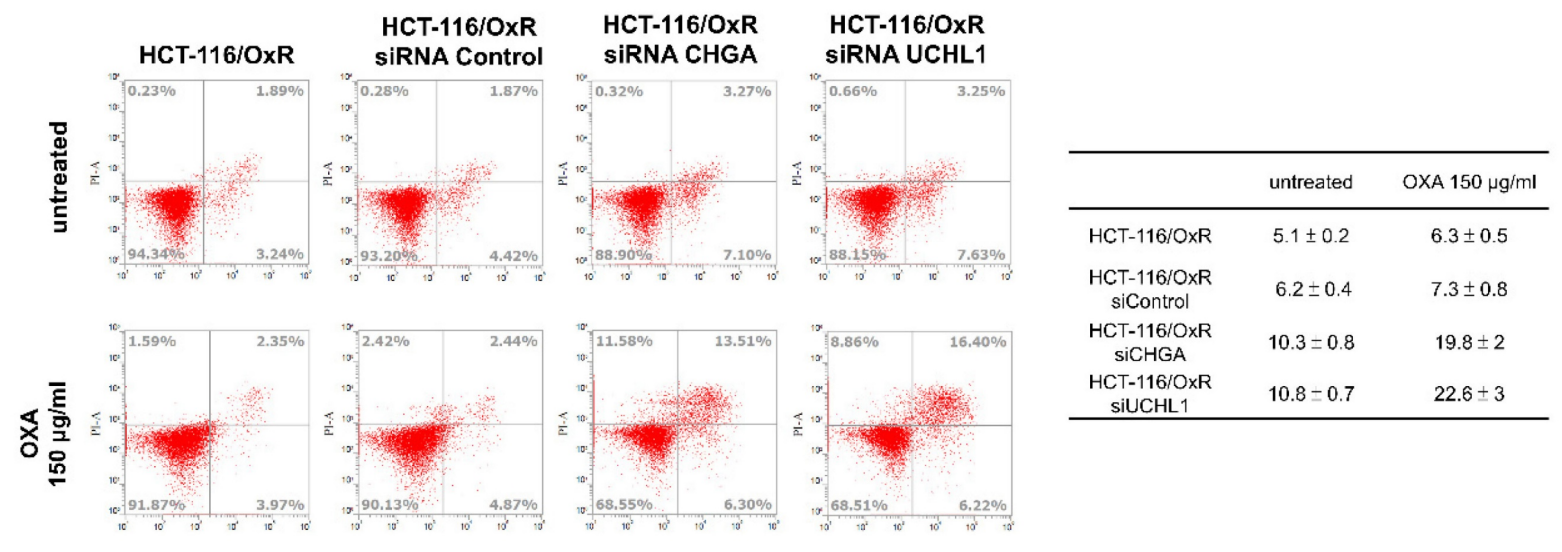
Silence analysis of ubiquitin carboxyl-terminal hydrolase isozyme L1 and chromogranin A expression on apoptosis in HCT-116/OxR with the addition of OXA to reduce drug resistance. Drug-resistant HCT-116/OxR cells underwent siRNA transfection procedures using siRNA Control, siRNA CHGA, and siRNA UCHL1. Medium replaced the transfection medium at the 6-hour, followed by OXA supplementation at 150 µg/ml. Flow cytometric evaluation commenced after Annexin V-PI staining application. Quantitative comparisons utilized HCT-116/OxR measurements as the reference standard, with baseline normalization set at 1 for subsequent sample analysis.

Similarly, calcium ions (Ca²⁺) are crucial cell signaling molecules that play key roles in physiological functions such as cell division, growth, survival, and transfer of signals. In the CHGA and UCHL1 siRNA treatment groups, calcium levels decreased to 0.7 ± 0.1 and 0.8 ± 0.1, respectively (Figure 4B). Additionally, in the cell cycle distribution experiments, the CHGA and UCHL1 siRNAs exhibited an increase in the proportion of HCT-116/OxR cells in the G1 phase to 41 ± 2% and 42 ± 2% (Figure 4C). These accumulations of cells in the G1 phase indicated that the knockdown of CHGA and UCHL1 causes cell cycle arrest, thereby reducing the proliferation ability of cancer cells. Next, the roles of CHGA and UCHL1 in drug-resistant CRC cells was explored, and whether inhibiting CHGA and UCHL1 elevated OXA sensitivity in the HCT-116/OxR. The analysis showed that the apoptosis rate of HCT-116/OxR was 5.1 ± 0.2%, whereas that of cells treated with siRNA CHGA or siRNA UCHL1 was 10.3 ± 0.8% and 10.8 ± 0.7%, respectively. When OXA 150 µg/mL was added, CHGA and UCHL1 siRNAs were shown to increase the rate of apoptosis in HCT-116/OxR cells to 19.8 ± 2%, and 22.6 ± 3% (Figure 5). Targeting these CHGA and UCHL1 siRNAs may lead to overcoming chemoresistance by inhibiting ROS, calcium signaling, and G1 phase dysregulation, and could restore chemosensitivity in HCT-116/OxR by disrupting survival mechanisms. These changes directly affected the growth of cancer cells by limiting proliferation ability, potentially inducing cell death and ER stress, as well as chemoresistance.

### Silencing of chromogranin A and ubiquitin carboxyl-terminal hydrolase isozyme L1 genes reverses chemoresistance by inhibiting Rho/ERK/NFκB-mediated epithelial mesenchymal transition in colorectal cancer

**Figure 6:**
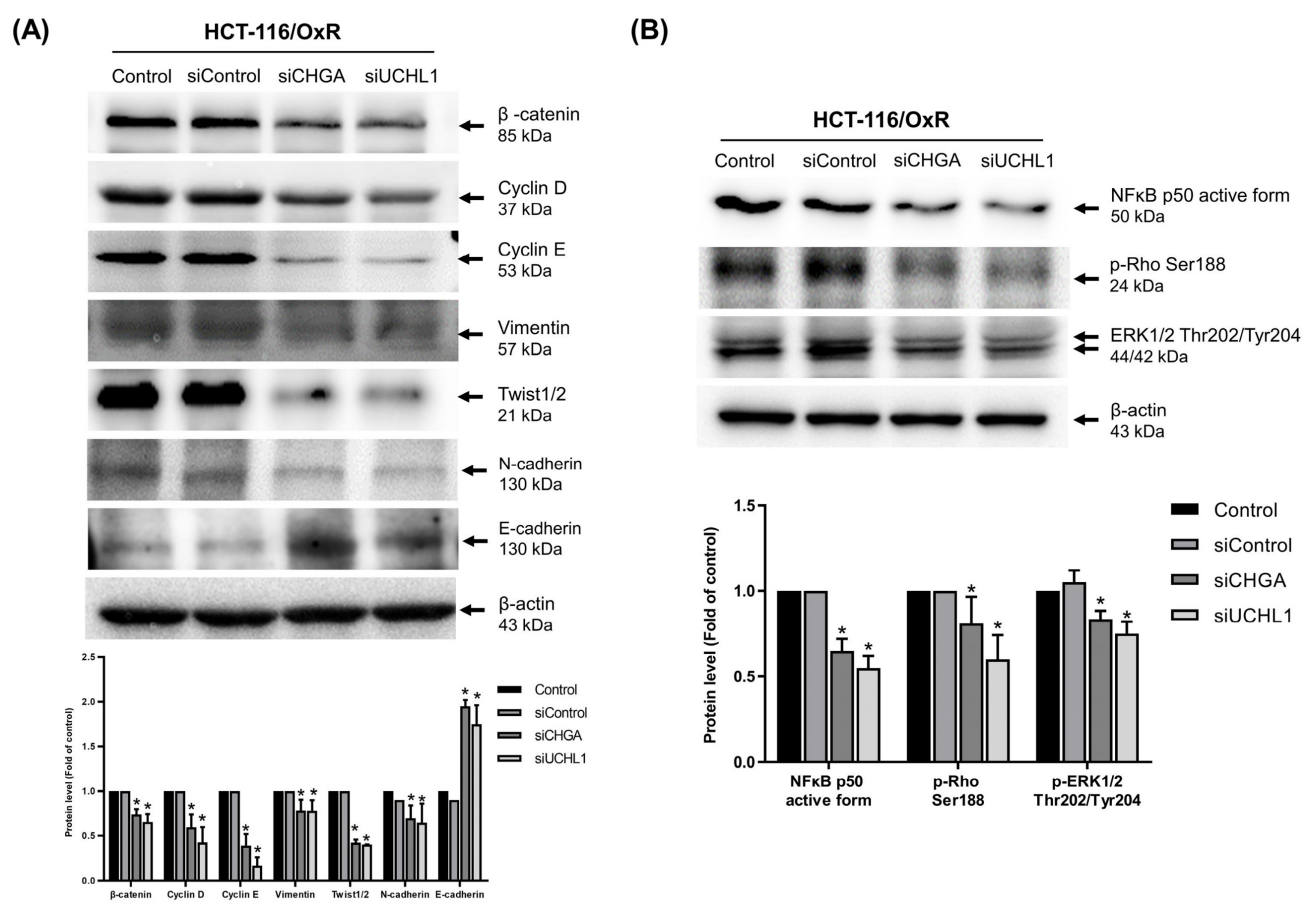
Investigation of how silencing CHGA and UCHL1 expression affects epithelial-mesenchymal markers and cell invasion through NFκB, ERK, and Rho pathways in oxaliplatin-resistant HCT-116/OxR cells. (A) Western blot analysis was performed to measure the changes in EMT-related marker proteins, including β-catenin, cyclin D, cyclin E, vimentin, and Twist, following the suppression of CHGA and UCHL1 expression. (B) The Rho/ERK/NFκB signaling pathway was analyzed under identical experimental conditions. β-actin was used as a loading control. Protein expression levels were quantified by densitometric analysis, with the untreated control group ratio normalized to onefold. Results are presented as mean ± SD, with statistical significance set at *p < 0.05.

The role of CHGA and UCHL1 in the chemoresistance mechanism of HCT-116/OxR cells was explored further. Previous studies have shown that the Rho/ERK/NFκB signaling pathway activates protein kinases, thereby inducing EMT and CSCs characteristics. Western blotting showed that siRNA-mediated silencing of the CHGA and UCHL1 genes significantly reduced the protein expression of multiple EMT-related markers, including β-catenin, cyclin D, cyclin E, vimentin, and Twist as well as N-cadherin, while siRNA CHGA and UCHL1 genes significantly increased the protein expression of E-cadherin (Figure 6A). Meanwhile, the expression of important signaling molecules, such as NF-κB, phosphorylated p-Rho, and phosphorylated p-ERK, also decreased significantly (Figure 6B). These findings verified that inhibiting CHGA and UCHL1 expressions effectively disrupts the invasive capacity of HCT-116/OxR chemoresistant CRC cells and related signaling pathways.

### Ablation of chromogranin A and ubiquitin carboxyl-terminal hydrolase isozyme L1 increases the efficacy of OXA and inhibits epithelial-mesenchymal transition capabilities in animal experiments

**Figure 7:**
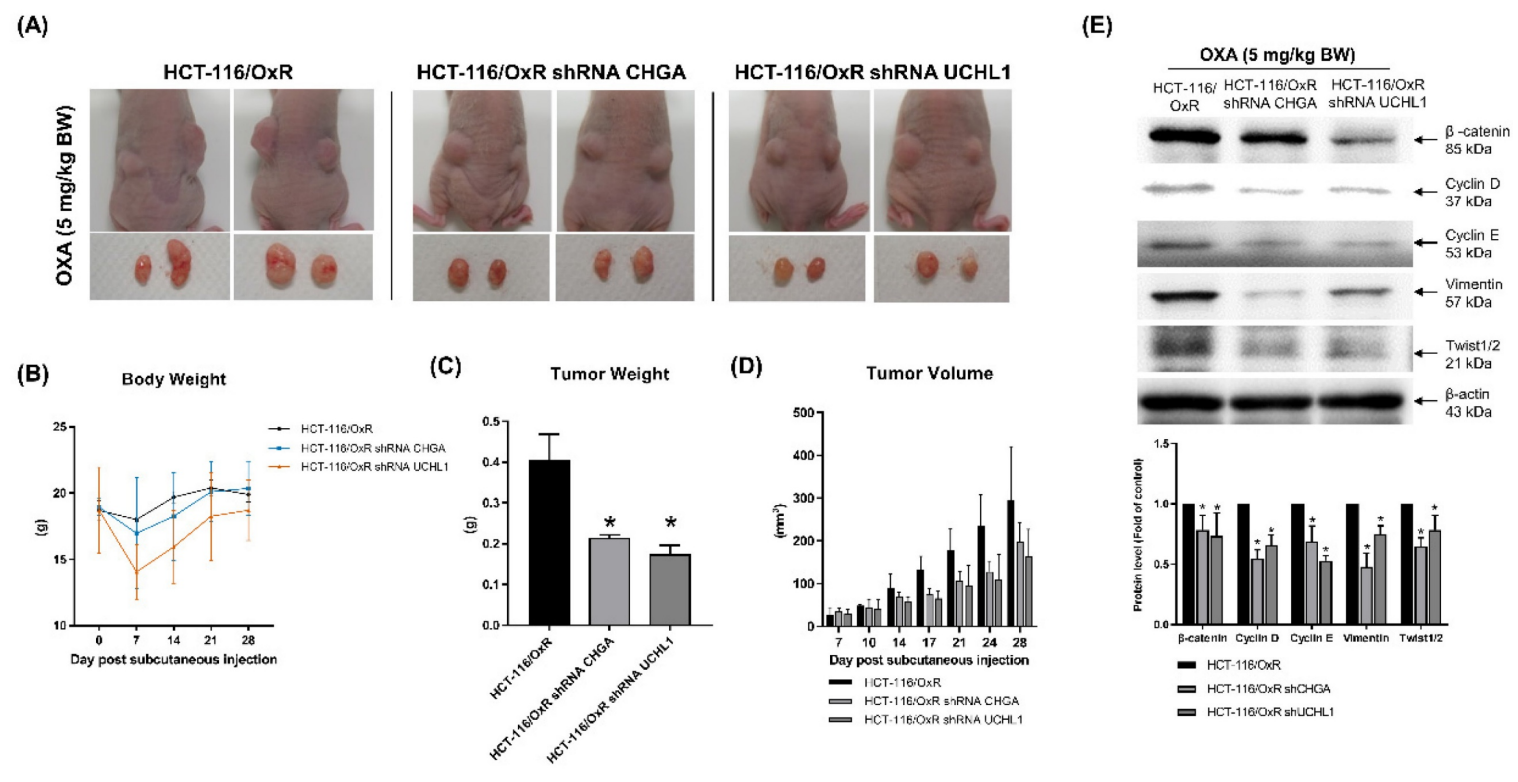
*In vivo*, the absence of CHGA and UCHL1 expression reduces the oxaliplatin resistance in HCT-116-116/OxR cells. Following the introduction of shRNA CHGA and shRNA UCHL1 into HCT-116/OxR resistant cells to achieve functional loss of both CHGA and UCHL1, injected the modified cells into mice for further investigation. Administered oxaliplatin at 5 mg/kg daily for five days post-injection to evaluate treatment effects. After euthanasia at study completion, conducted detailed analysis of: (A) tumor growth size, (B) statistical documentation of mouse weight, (C) extracted tumor mass, and (D) comparative analysis of tumor volume. The data are presented as means ± SD. (E) Excised tumor tissues from *in vivo* models of drug-resistant HCT-116/OxR carcinoma demonstrating absence of CHGA and UCHL1 expression were processed for molecular characterization. Immunoblot analysis was performed to evaluate the expression profile of β-catenin, Cyclin D/E, Vimentin, and Twist. β-actin immunoreactivity was employed as the loading reference standard. Relative protein abundance was determined via densitometric quantification, with baseline expression in control specimens normalized to unity. Numerical data are expressed as mean values ± SD, with statistical significance indicated at *p < 0.05.

Analysis of cell experiment data verified that reducing the expression of CHGA and UCHL1 affects the intracellular properties of chemoresistant CRC cells, HCT-116/OxR. To further verify these findings and the *in vivo* effects, animal experiments were conducted. In the experiment, HCT-116/OxR shControl cells and shRNA CHGA and shRNA UCHL1 HCT-116/OxR cells were injected into the nude mice. OXA treatment (5 mg/kg) was administered for 5 consecutive days, and subsequent changes were observed. On day 21 of the experiment, the research results showed that compared with the untreated HCT-116/OxR shControl group, the tumors in the experimental group transfected with shRNA CHGA and shRNA UCHL1 (Figure 7A) were significantly reduced. The body weight of mice in this group was slightly lower than that of mice in the shControl group (Figure 7B). Statistical data further showed a reduction in tumor weights in the experimental group to 50% and 42% of those in the shControl group, respectively. Tumor volume was significantly smaller than that in the HCT-116/OxR shControl group (Figure 7C and D). Cell and animal experiments showed that inhibition of the expression of CHGA and UCHL1 oncogenes effectively reduced the malignant manifestations of cancer cells. Our study demonstrates that downregulation of CHGA and UCHL1 genes effectively inhibits growth potential in the OXA-resistant CRC cell line HCT-116/OxR through suppression of the Rho/ERK/NFκB signaling pathway. Laboratory experiments with animal models confirmed that specifically targeting these molecular markers significantly enhances the therapeutic effectiveness of OXA. Western blot analysis of extracted tumor tissues examining EMT regulators (including β-catenin, cyclins D/E, vimentin, and Twist transcription factor) revealed a remarkable pattern. We observed that shRNAs CHGA and UCHL1 consistently resulted in a substantial reduction of mesenchymal characteristics across the analyzed tumor specimens (Figure 7E). These findings suggest that disruption of EMT processes enables better penetration of chemotherapy drugs and increased cancer cell death, leading to clinically relevant reduction in tumor size and improved treatment response indicators.

### Expression of chromogranin A and ubiquitin carboxyl-terminal hydrolase isozyme L1 were regulated by NFκB/ERK/Rho-mediated epigenetic histone modification in HCT-OxR cells

**Figure 8:**
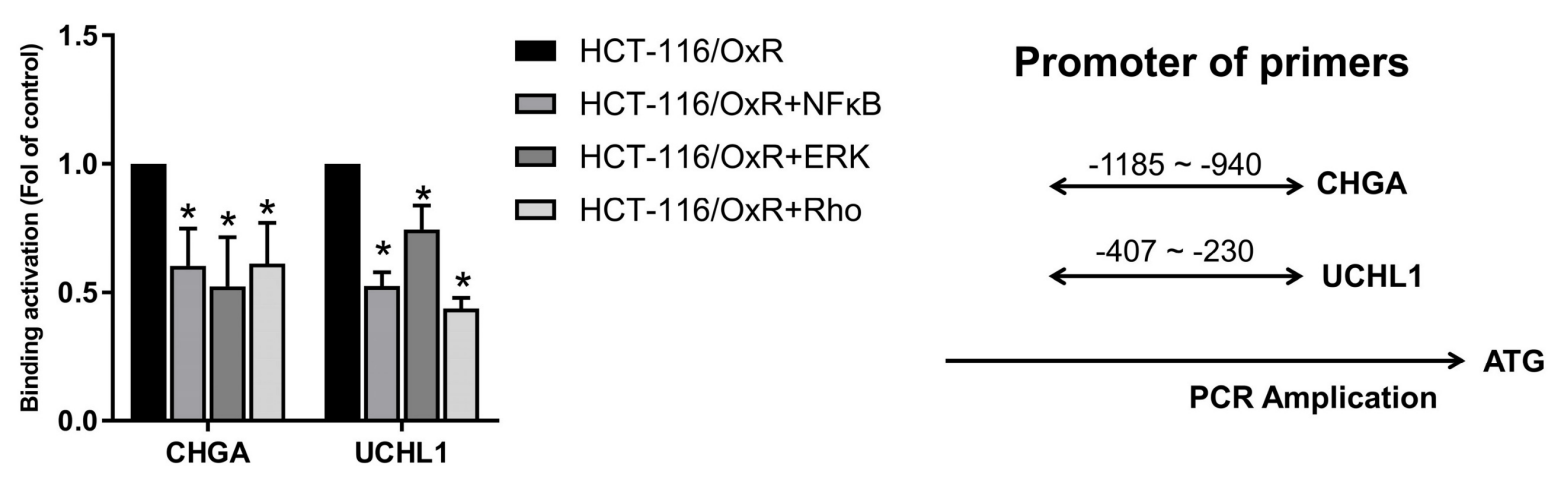
ChIP-qPCR analysis of H3K4me3 at CHGA and UCHL1 promoters following pathway inhibition in HCT-OxR cells. HCT-OxR cells were treated with specific inhibitors targeting the NF-κB, ERK, or Rho signaling pathways. Chromatin immunoprecipitation (ChIP) was performed using an antibody against H3K4me3, followed by quantitative PCR (qPCR) to measure H3K4me3 enrichment at the CHGA and UCHL1 promoter regions. Results are expressed as fold enrichment relative to the untreated HCT-OXR group (set as 1.0 fold). Data represents the mean ± SD. * p < 0.05, as compared to the untreated control group.

Multiple signaling molecules induce cell apoptosis through the H3K4me3 of histone H3 (30,31). Then, we attempted to study whether CHGA and UCHL1 could mediate the signaling pathways and regulate the EMT process through NFκB/ERK/Rho, which were related to the regulation of histone H3 lysine 4 trimethylation (H3K4me3) histone modification on the promoter regions of the CHGA and UCHL1 genes. (16,33). This chromatin immunoprecipitation assay demonstrates the effects of inhibitors for RhoGTPase (CCG-1423 (1.0 μM)), ERK (PD98059 (10 μM)), and NF-κB (PDTC (50 μM)) on the H3K4me3 trimethylation of binding promoter regions of CHGA and UCHL1 genes in HCT-116/OxR cells. For CHGA gene expression, compared with the untreated control group (HCT-116/OxR, set at 1.0 fold), treatment with Rho, ERK, and Rho inhibitors reduced the binding activity to approximately 60%, 52%, and 61%, respectively. Considering the UCHL1 gene expression, Rho, ERK, and Rho inhibitors decreased the epigenetic histone modification of binding activity to approximately 42%, 75%, and 52% (Figure 8). These results indicate that NFκB/ERK/Rho signaling pathways regulated the expression of CHGA and UCHL1 for H3K4 methylation and transcription activation involved in OXA-resistant CRC HCT-116/OxR survival and metastasis.

## Discussion

CRC is a globally aggressive malignancy where drug resistance compromises treatment efficacy across all modalities, resulting in lowered survival outcomes 34,35. Several resistance mechanisms and tumor complexity increase treatment duration and costs 36,37, necessitating innovative personalized approaches for improved outcomes. Previous investigations revealed CHGA and UCHL1 as critical mediators of lymph node metastasis in CRC, functioning through the H3K4 trimethylation-regulated Rho/NFκB signaling 16. Our study investigated the unexplored mechanisms by which CHGA and UCHL1 protein expression contribute to EMT induced by OXA resistance. MTT assays revealed a significantly higher IC_50_ of HCT-116/OxR cells (150 μg/mL) than that of HCT-116 CRC cells IC_50_ (20 μg/mL). When treated with OXA (10 & 20 μg/mL) (Figure 1). CHGA and UCHL1, known oncogenic factors in various cancers 38,39, were investigated for their role in drug resistance through siRNA silencing in HCT-116/OxR cells. Previous studies have shown that CHGA drives CRC progression by increasing tumor invasion depth and lymph node metastasis, reducing survival rates. CHGA High level tumors show greater aggressiveness and worse outcomes. Other studies indicated that CHGA-positive neuroendocrine cells secrete substances that enhance the proliferation and invasion of the surrounding cancer cells through autocrine/paracrine signaling 40. UCHL1 drives malignant progression in cancer. Highly expressed in tumor tissues, UCHL1 correlates with poor prognosis and advanced disease; its deubiquitinase activity promotes angiogenesis, which enhances cellular proliferation and migration. Functional studies confirm the essential role of UCHL1 in tumor growth and metastatic potential 41. For a deeper understanding of this phenomenon, we conducted additional experiments, which demonstrated that reducing CHGA and UCHL1 expression decreased the invasiveness and metastatic potential of drug-resistant HCT-116/OxR cells (Figure 2, Figure 3). The research further revealed that the knockdown of CHGA and UCHL1 genes led to reduced reactive oxygen species generation and calcium ion concentrations, while simultaneously inducing cell cycle arrest in the G1 phase. These findings indicated that the suppression of CHGA and UCHL1 interferes with cell cycle progression, thereby inhibiting cellular proliferation capabilities (Figure 4). siRNA-mediated knockdown of CHGA and UCHL1 sensitized drug-resistant HCT-116/OxR cells to OXA, evidenced through increased cell death rates measured through Annexin V-PI staining, demonstrating that suppression of CHGA and UCHL1 genes reduces chemoresistance (Figure 5).

Building on prior research identifying CHGA and UCHL1 as critical components of the Rho/ERK/NFκB pathway 41,42, our findings demonstrate the essential role of these proteins in promoting chemoresistance and survival in OXA-resistant CRC. Silencing CHGA and UCHL1 significantly reduced invasive potential, reversed EMT, and restored OXA sensitivity. These findings highlight the therapeutic potential of targeting CHGA and UCHL1 to overcome drug resistance in CRC. Further research through *in vivo* models is needed to explore their regulatory dynamics and investigate precise molecular interactions with downstream effectors. Mechanistically, both proteins can alter the secretion of cytokines and growth factors, reshaping the tumor microenvironment to support metastatic spread. Specific mechanisms linking ER stress to chemoresistance through CHGA and UCHL1 regulation involve several interconnected pathways. ER stress activates the unfolded protein response (UPR), which upregulates CHGA and UCHL1 expression. CHGA, as a secretory granin protein, modulates calcium homeostasis and vesicular trafficking, potentially sequestering oxaliplatin and reducing its cytotoxicity. UCHL1, a deubiquitinating enzyme, likely stabilizes anti-apoptotic proteins and promotes pro-survival autophagy under ER stress conditions. Previous studies have shown demonstrated UCHL1 upregulation in cisplatin-resistant ovarian cancer cells following ER stress. Similarly, Xu *et al.* reported CHGA involvement in chemoresistance through calcium signaling modulation in pancreatic cancer. These findings parallel our observations in oxaliplatin-resistant colorectal cancer. The proposed mechanism involves ER stress triggering UPR activation, leading to CHGA/UCHL1 upregulation, which subsequently activates Rho/ERK/NFκB signaling and promotes EMT. This cascade enhances cell survival, migration, and invasion while reducing oxaliplatin-induced apoptosis. Pharmacological targeting could focus on inhibiting UPR sensors (PERK, IRE1α), directly suppressing CHGA/UCHL1, or blocking downstream Rho/ERK/NFκB signaling to overcome resistance. Targeting the Rho/ERK/NF-κB signaling pathway may enhance sensitivity to oxaliplatin by disrupting survival mechanisms. Inhibiting CHGA could reduce angiogenesis and immune evasion, while targeting UCHL1 may promote the degradation of oncogenic proteins, leading to increased apoptosis. Furthermore, combining oxaliplatin with inhibitors that disrupt this signaling network could enhance treatment efficacy and overcome resistance, presenting a promising strategy for improving therapeutic outcomes in chemoresistant colorectal cancer (CRC). Evaluating combination therapies that target these pathways alongside standard chemotherapeutics will be essential for developing effective clinical interventions to improve outcomes for patients with chemoresistant CRC.

Our investigation into CRC drug resistance revealed that siRNA CHGA and siRNA UCHL1 in OXA-resistant HCT-116/OxR cells significantly diminished expression of EMT markers (β-catenin, cyclin D/E, vimentin, Twist) and critical signaling proteins (Rho/ERK/NF-κB), as confirmed (Figure 6A & 6B). The above experimental results confirm that drug-resistant CRC cells activate the Rho/ERK/NFκB pathway, inducing EMT. CHGA and UCHL1 are critical for invasion and metastasis in these cells, with their silencing disrupting these processes. HCT-116/OxR-resistant cells showed elevated ROS levels compared to the siRNA CHGA and siRNA UCHL1 groups. Silencing these genes resulted in G1 phase arrest, reduced G2 phase proportion, and decreased cyclin D/E levels, indicating lower proliferative capacity. These findings align with the known roles of microRNAs (miRNAs) in cancer progression. Being critical non-coding RNA molecules, miRNAs play key roles in cancer initiation and progression 43 by binding to target mRNAs and regulating gene expression, thereby impacting cellular processes, including proliferation, differentiation, apoptosis, and migration 44. In cancer, the expression of miRNAs frequently exhibits dysregulation, with certain miRNAs being downregulated as tumor suppressors while others are upregulated as oncogenes 45,46. For instance, research demonstrates that miR-29a-3p is highly expressed in CRC and confers OXA resistance in cancer cells 47. Additionally, similar studies have indicated that dysregulation of miRNA leads to the development of drug resistance 48. Furthermore, miRNAs modulate cancer cell metabolic reprogramming, angiogenesis, and immune evasion, and even play a role in shaping the tumor microenvironment 49-52. While this experiment primarily investigates the roles of CHGA and UCHL1 in OXA-resistant CRC, the potential involvement of miRNAs in regulating these genes and their associated pathways represents a promising direction for future research.

CHGA and UCHL1 have significant value as predictive proteins for cancers, with research showing that their expression levels in CRC tissues differ markedly from those in normal tissues, as potential diagnostic markers 52-54. In this study, we established cell lines of HCT-116/OxR cells using CHGA and UCHL1 shRNAs and evaluated their biological roles in cell invasion, survival, and EMT-associated markers. Reduced levels of CHGA and UCHL1 effectively inactivated the Rho/ERK/NFκB pathway while simultaneously downregulating β-catenin, cyclin D/E, vimentin, and Twist in HCT-116/OxR cells, confirmed both *in vitro* and in mice* in vivo* (Figure 6,7,8). Notably, transfection with shRNAs targeting CHGA and UCHL1 significantly restored chemosensitivity in previously resistant cells. When HCT-116/OxR cells, which had silenced CHGA and UCHL1, were treated with OXA, they exhibited markedly increased drug sensitivity compared with control resistant cells, with enhanced apoptotic responses and reduced cell viability. In animal models, these transfected HCT-116/OxR cells treated with OXA led to the development of significantly smaller tumors compared to untransfected drug-resistant HCT-116/OxR cells, with tumor weights reduced by 50% and 42% respectively, along with decreased tumor volume. These findings demonstrate that reducing CHGA and UCHL1 expression not only inhibits cellular carcinogenesis in CRC cells and animal models (Figure 7) but also reverses chemoresistance effectively, suggesting a promising therapeutic strategy for overcoming drug resistance in CRC.

H3K4me3 facilitates carcinogenesis, disease development, and cancer-associated functions in CRC. Beyond its involvement in transcriptional regulation, repair mechanisms, replication processes, and transcription-complex protein interactions, it additionally modulates the Rho-GTPase/ERK/NF-κB signaling cascades, thereby inducing cellular proliferation, migratory behavior, differentiation processes, and resistance to chemotherapy 16,55,56 This epigenetic regulation presents potential targets for cancer therapy. Experimental evidence demonstrates that treatment with inhibitors alters H3K4me3-mediated transcriptional activation at CHGA and UCHL1 promoter regions. In HCT-116/OxR cells, these two genes, CHGA and UCHL1, are differentially regulated through distinct signaling cascades: via the Rho/ERK/NF-κB pathway, both involving H3K4 methylation of their respective promoters (Figure 8). Treatment with specific inhibitors (CCG-1423 for Rho, PD 98059 for ERK, and PDTC for NF-κB) demonstrated pathway-specific effects: the expression of CHGA and UCHL1 genes was suppressed by all three inhibitors. Furthermore, the Wnt/β-catenin signaling pathway emerges as a potential master regulator in these cells, capable of transcriptional upregulation of both CHGA and UCHL1. Through this hierarchical regulation, these genes function as novel mediators of cell survival and metastatic pathways, ultimately influencing the formation of the tumor microenvironment. Future research should focus on comprehensive validation of this Wnt/β-catenin-mediated regulation through multiple complementary approaches. Additionally, pharmacological modulation of the pathway using specific inhibitors at different nodes could provide mechanistic insights into how the Wnt signaling orchestrates chemoresistance through CHGA and UCHL1 regulation. These multifaceted approaches are essential for fully elucidating the roles of these genes in OXA-resistant cell growth and potentially identifying novel therapeutic vulnerabilities that could be exploited to overcome chemoresistance.

## Conclusions

**Figure 9:**
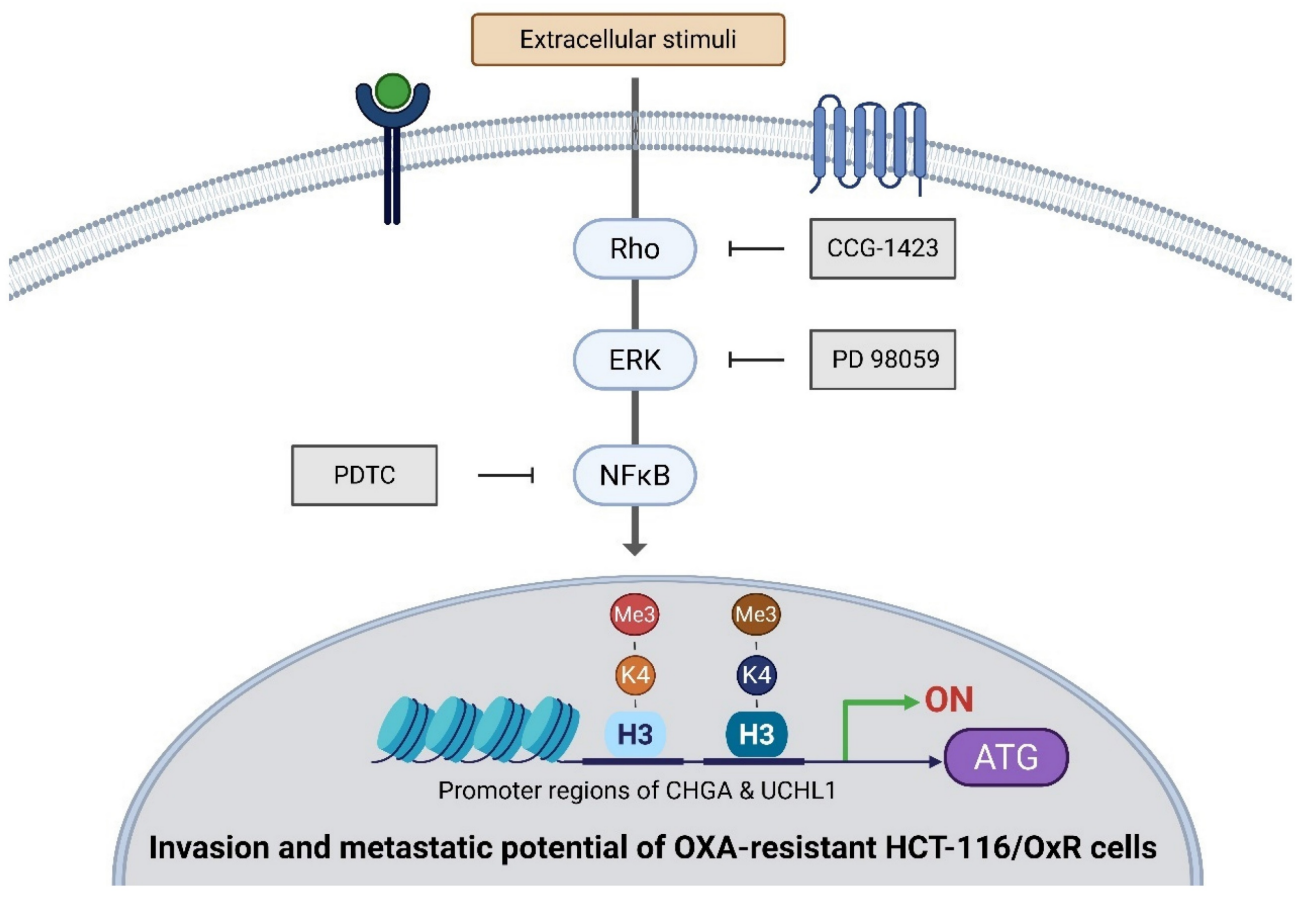
Diagrammatic representation of the operational mechanism through which CHGA and UCHL1 contribute to the enhancement of oxaliplatin resistance in HCT-116/OxR cells via intrinsic Rho-GTPase/ERK/NFκB signalling cascades that modulate histone modifications (specifically H3K4me3), establishing these factors as dependable potential biomarkers associated with chemotherapeutic resistance.

This study reveals significantly elevated expression of CHGA and UCHL1 is in CRC tissues undergoing recurrence and chemoresistance, suggesting their potential role as biomarkers for metastatic CRC. Both the discussed proteins contribute to drug resistance in HCT-116/OxR cells, particularly through the Rho/ERK/NFκB signaling pathway, and their expression is epigenetically regulated by H3K4 methylation at their respective promoter regions. Specifically, CHGA and UCHL1 were found to be regulated through Rho/ERK/NF-κB pathways, both dependent on transcriptional activation mediated by H3K4me3. The inhibition of CHGA and UCHL1 expression reduces the invasion and metastatic potential of drug-resistant CRC cells, slows cellular proliferation, and diminishes drug resistance. These findings highlight the significance of CHGA and UCHL1 in CRC progression and their potential as therapeutic targets, concurrently suggesting that targeting their epigenetic histone modification might provide additional therapeutic strategies (Figure 9).

## Data Availability

All relevant data are within the paper. The raw data supporting the conclusions of this manuscript will be made available by the authors, without undue reservation, to any qualified researcher.

## Abbreviations

CHGA: Chromogranin A
CRC: Colorectal cancer
CSC: Cancer stem cells
EMT: Epithelial-mesenchymal transition
miRNAs: microRNAs
OXA: Oxaliplatin
TCF: T-cell factor
UCHL1: Ubiquitin carboxy-terminal hydrolase L1
